# Populations of NK Cells and Regulatory T Cells in the Endometrium of Cycling Mares—A Preliminary Study

**DOI:** 10.3390/ani12233373

**Published:** 2022-11-30

**Authors:** Joanna Jaworska, Amanda M. de Mestre, Joanna Wiśniewska, Bettina Wagner, Arkadiusz Nowicki, Ilona Kowalczyk-Zięba, Izabela Wocławek-Potocka

**Affiliations:** 1 Department of Gamete and Embryo Biology, Institute of Animal Reproduction and Food Research, Polish Academy of Sciences, 10-747 Olsztyn, Poland; 2 Department of Comparative Biomedical Sciences, Royal Veterinary College, Hertfordshire, Hatfield AL9 7TA, UK; 3 Laboratory of Cell and Tissue Analysis and Imaging, Institute of Animal Reproduction and Food Research, Polish Academy of Sciences, 10-747 Olsztyn, Poland; 4 Department of Population Medicine and Diagnostic Sciences, College of Veterinary Medicine, Cornell University, Ithaca, NY 14853, USA; 5 Private Veterinary Practice Arkadiusz Nowicki, 10-684 Olsztyn, Poland

**Keywords:** Tregs, uterus, horse, fertility, reproduction

## Abstract

**Simple Summary:**

Endometrial immune cells, such as regulatory T cells and NK cells, are not well known in mares. Nevertheless, these cells play important roles both in physiological and pathological processes in equine reproduction. This work aimed to characterize populations of regulatory T cells and NK cells in the endometrium of cycling mares. With the use of specific antibodies, we showed that two different types of regulatory T cells and NK cells are present both during estrus and diestrus in mares.

**Abstract:**

Endometrial immune cells are essential to support uterine functions across the estrous cycle and in preparation for pregnancy. It has been acknowledged that changes in phenotype and/or numbers of lymphocytes, such as regulatory T cells (Tregs) and NK cells, might result in lower fertility in women and mice. Little is known about equine endometrial immune cells across the estrous cycle. Here, we compared the populations of endometrial Tregs and NK cells in estrus and diestrus in mares. Endometrial biopsy and blood samples were taken in estrus and diestrus from 11 mares ages 4–12 years. Flow cytometry with anti-CD4, -CD25 and -FOXP3 and anti-NKp46 and -CD3 antibodies was used to determine the populations of Tregs and NK cells, respectively. The concentration of progesterone was measured with chemiluminescence immunoassay. The results were analyzed with paired Student *t* tests. The mean percentage of endometrial CD4^+^FOXP3^+^ Tregs was 13.7 ± 6.2% in diestrus and 14.5 ± 5.9% in estrus, while the mean percentage of endometrial CD4^+^FOXP3^+^CD25^+^ Tregs changed from 3.6 ± 2.1% in diestrus to 2 ± 2% in estrus (*p* = 0.0947). The mean proportion of CD3^−^NKp46^+^ lymphocytes in the endometrium was not significantly different, with 6 ± 1% in estrus and 6.5 ± 1.4% in diestrus. There was a large variation in the percentage of NK cells between mares of 2.1–12.7%. This study showed, for the first time, the presence of CD4^+^FOXP3^+^CD25^+^ Tregs and CD3^−^NKp46^+^ NK cells in the endometrium of non-pregnant cycling mares. The percentage of Tregs, and to a greater extent NK cells, showed large fluctuations between mares. Both Tregs and NK cells might be important for the preparation of the endometrium for semen deposition and pregnancy; however, further research is required.

## 1. Introduction

Uterine immune cells have two important functions. They protect the uterus against pathogens and prepare the endometrium for placentation and maternal–fetal tolerance during pregnancy [1,2]. Leukocytes present in the uterus comprise T cells, macrophages, dendritic cells, natural killer cells (NK cells) and mast cells [1,3]. Every leukocyte subset may have different physiological roles depending on their phenotype, and the abundance of these cells may vary between species due to differences in the anatomy of the endometrium, placenta and/or duration of pregnancy. During the reproductive cycle, the uterine environment undergoes cyclic changes which are influenced by hormones, such as progesterone and estrogens [1,2]. These hormones induce the expression of chemokines, cytokines and various receptors, which in turn may influence the recruitment and/or accumulation of specific leukocyte subsets [4,5]. Depending on the phase of the reproductive cycle, immune cells are involved in the preparation of the uterine environment for semen deposition and later the implantation of the embryo [5].

There are limited studies describing the phenotype and abundance of immune cells in the endometrium across the estrous cycle in the mare. Current data suggest that both CD4^+^ and CD8^+^ T cells are present in the endometrium and their numbers do not change across the cycle. However, their abundance may vary between mares [6,7], and subsets of these T cells are not defined. The majority of research on endometrial leukocytes has focused on the pregnancy period, and it is known that on days 35–45 of gestation, maternal CD4^+^ and CD8^+^ lymphocytes accumulate around the invading trophoblast. Interestingly, even though the trophoblast expresses high levels of fetal MHC I, trophoblasts are not destroyed by the maternal immune cells. Expressions of markers specific for the NK cells, NKp46, CD16, CD56 and CD94, have been detected in the lymphocytes surrounding the trophoblast [8]. NK cells are characterized by the lack of CD3 molecules; however, in studied species, the expression of other receptors, such as cytotoxicity and adhesion receptors, differ between peripheral and uterine NK cells (uNK) [4], resulting in species variation in phenotype and function. Uterine NK cells are suggested to participate in the preparation of the uterine immune environment for the embryo implantation in women [4,5]. It is hypothesized that in mares, uNK cells may control the invasion of the trophoblasts [8]; however, research on this subject is missing. CD4+ T cells expressing FOXP3, a transcription factor determining immunosuppressive properties of T cells, have been isolated from equine endometrium in the region of invasive trophoblasts of the endometrial cups [9]. These cells are considered regulatory T cells (Tregs) and are suggested to be involved in the maternal–fetal tolerance. In humans and mice, Tregs are usually defined as CD4^+^CD25^+^FOXP3^+^ cells; nonetheless, for these species, other markers specific for different Tregs lineages have also been described, reviewed in [10,11]. Peripheral Tregs with the CD4^+^CD25^+^FOXP3^+^ phenotype have been reported in horses [12]. CD25 receptor on Tregs binds interleukin 2 (IL-2), a cytokine, which is involved in the proliferation, differentiation and functioning of Tregs [10]. In other species, Tregs are suggested to be sparse in the endometrium over the estrous cycle; nonetheless, their numbers increase during pregnancy, most likely in response to paternal/fetal antigens [9]. Interestingly, a study by Kallikourdis and Betz [13] reported that in mice, endometrial FOXP3 mRNA levels are higher in estrus in comparison to diestrus. Whether the same phenomenon is present in mares has not been studied.

Studies on humans and mice showed that alterations in the populations of endometrial immune cells, specifically uNK and Tregs, may lead to reproductive problems such as infertility and miscarriage [5]. Immune cells present before embryo implantation are essential in preparation of the endometrium for a successful pregnancy [5,9]. Up to 8% of mares experience early pregnancy loss, that is, up to day 65 [14]. Worth noting is the fact that approximately 60% of early pregnancy losses in mares have unknown etiology [14,15]. Nevertheless, the studies on the endometrial lymphocytes which may be involved in the preparation of the uterus for pregnancy in mares are limited.

The aim of this study was to characterize equine endometrial NK cells and Tregs at two stages of the cycle, estrus and diestrus. Samples were taken during late estrus to reflect the stage of the cycle when the endometrium is prepared for the presence of semen and estrogen blood concentrations are high, and during mid diestrus when the progesterone blood concentration is high, and, in the case of pregnancy, the embryo descends to the uterus. We hypothesized that both Tregs and NK cells will be present in the endometrium of non-pregnant cycling mares and the percentage of these cells will differ with regard to the phase of the estrous cycle. In addition, the phenotypes of these cells were characterized using the available antibodies.

## 2. Materials and Methods

### 2.1. Animals

Eleven mares of mixed breeds ages 4–12 years were used in the study. Six mares had at least one previous pregnancy and five mares had no previous pregnancies. All procedures were approved by the Local Ethical Committee in Olsztyn (approval number 72/2021). Prior to the study, all mares were examined by a veterinarian and found to be clinically healthy. Mares were monitored with the use of transrectal ultrasonography to determine the day of ovulation, which was considered as day 0 of the estrous cycle. Endometrial biopsies (2–3 biopsies per mare) were collected during diestrus and estrus. Diestrus was defined as the presence of a corpus luteum (CL) on one of the ovaries and the absence of uterine edema as determined by transrectal ultrasound examination, a peripheral progesterone concentration of ≥4 ng/mL and confirmation of ovulation 7 to 9 days prior to sampling. Estrus was defined as the presence of a follicle ≥35 mm and uterine edema as determined by transrectal ultrasound examination together with a peripheral progesterone concentration of <1 ng/mL. Briefly, sterile alligator jaw biopsy forceps were introduced through the vulva and cervix, and biopsy of the endometrium was taken from the base of one of the uterine horns. Immediately after collection, the samples were placed in phosphate-buffered saline (PBS, Merck, Darmstadt, Germany) supplemented with 0.5% fetal calf serum (FCS, Merck, Darmstadt, Germany) and transported to the laboratory at 4 °C. To determine the concentration of progesterone (P4), blood samples were taken from a jugular vein in 10 mL tubes containing increased silica act clot activator (BD Vacutainer, Franklin Lakes, NJ, USA) at the time of each biopsy collection. Blood samples were centrifuged at 2000× *g* for 15 min at RT and serum was collected and immediately frozen at −20 °C until further analysis.

### 2.2. Tissue Preparation

Lymphocyte suspensions were obtained as described before [10]. In brief, tissue was minced and digested in RPMI medium with 25 mM Hepes (Sigma, St. Louis, MO, USA), 1% FCS, 1% (*w*/*v*) bovine serum albumin (Sigma, St. Louis, MO, USA), 35 U/mL DNase (Sigma, St. Louis, MO, USA) and 200 U/mL collagenase Type I (Sigma, St. Louis, MO, USA) and incubated at 37 °C for 45 min in total. Cell suspensions passed through 100 μm and then 40 μm cell strainers (Merck, Darmstadt, Germany), washed in PBS/0.5% FCS and subjected to density gradient centrifugation with the use of Ficoll-Paque Plus (Merck, Darmstadt, Germany). Cell suspensions enriched in lymphocytes were washed twice in PBS/0.5% FCS. The viability of isolated cells was evaluated with Trypan Blue and a hemocytometer and ranged between 80% and 90% for all samples. The number of cells isolated per g of tissue ranged from 1 to 2 × 10^6^.

### 2.3. Flow Cytometry

**Figure 1:**
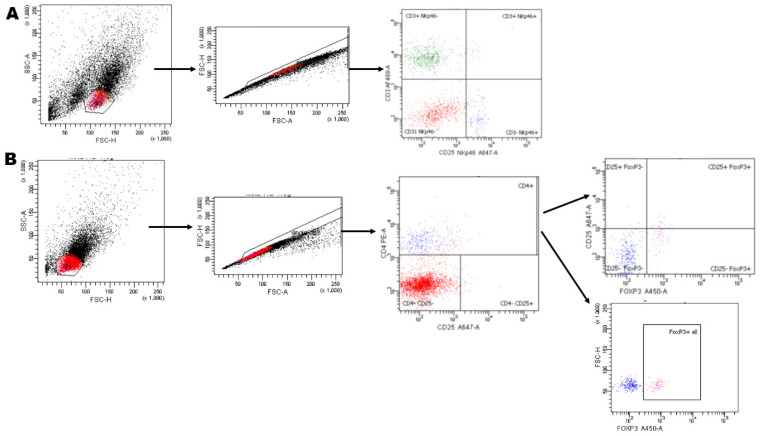
Gating strategy for NK cells (**A**) and regulatory T cells (Tregs) (**B**) present in the endometrium of cycling mares. (**A**) Gate was set for the lymphocytes (forward and side scatter); cells then gated to exclude cell debris and doublets; based on the fluorescence signal populations of lymphocytes expressing (or not), CD3 and NKp46 (CD3^−^NKp46^+^ lymphocytes) were determined. (**B**) Gate was set for the lymphocytes (forward and side scatter); cells gated to exclude cell debris and doublets; next, the gate was positioned on CD4^+^ cells; based on the fluorescence signal within the CD4^+^ lymphocytes’ gated population, cells expressing FOXP3 alone (representing CD4^+^FOXP3^+^ lymphocytes) or FOXP3 and CD25 (representing CD4^+^CD25^+^FOXP3^+^ lymphocytes) were determined.

Antibodies determining the phenotype and presence of Tregs and NK cells were chosen based on previous literature (Table 1). Flow cytometry analysis was performed separately for NK cells and Tregs. To detect Tregs, immediately after isolation, lymphocytes were labeled with anti-CD4 and anti-CD25 antibodies. Fixation, permeabilization and staining with anti-FOXP3 antibody (Table 1) was performed with a FOXP3 staining kit (Invitrogen, CA, USA) according to the manufacturer’s instructions. For the NK cells, isolated cells were labeled with anti-CD3 and anti-NKp46 antibody. Previous reports showed that NKp46 receptor, which is a natural cytotoxicity receptor and is conserved among species [16], is expressed mainly in the CD3-depleted population of lymphocytes [17]; thus, the anti-CD3 antibody was used as a negative marker of NK cells in our study. Immunofluorescence flow cytometry was performed using BD FACSAria II Cell Sorter (Becton Dickinson, Franklin Lakes, NJ, USA). There were 10,000 counts acquired per sample, and 8000 counts were included in the generated data for every sample. BD FACSDiva Software v. 6.1.3 was used to analyze the data. An example of the representative strategy used for FACS analysis is shown in Figure 1.

### 2.4. Progesterone (P4) Concentrations

P4 concentration in peripheral blood serum was measured using the chemiluminescence immunoassay (CLIA) method with the use of Immulite 2000 Xpi (Siemens, Munich, Germany), as directed by the manufacturer. The detection range was 0.2–40 ng/mL.

### 2.5. Statistical Analysis

The distribution of data was tested with D’Agostino Pearson and Shapiro–Wilk tests. To compare the percentage of Tregs and NK cells between mares in estrus and diestrus, paired two-tailed Student’s t tests were used. For every test, alpha error was set at 5%, and analysis was performed using GraphPad Prism software (v.9.0, San Diego, CA, USA).

## 3. Results

### 3.1. NK Cells

**Figure 2:**
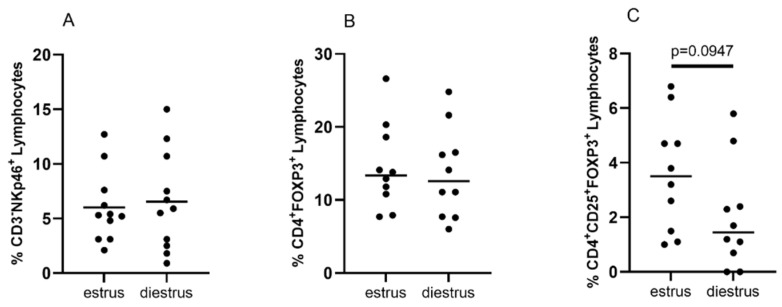
Comparison of the percentage of CD3^−^NKp46^+^, CD4^+^FOXP3^+^ and CD4^+^CD25^+^FOXP3^+^ endometrial lymphocytes between estrus and diestrus in 11 mares. There were no significant differences between CD3^−^NKp46^+^ lymphocytes and CD4^+^FOXP3^+^ lymphocytes in estrus and diestrus (*p* > 0.05 (**A**,**B**). The percentage of CD4^+^CD25^+^FOXP3^+^ lymphocytes was higher in estrus than diestrus (*p* = 0.0947) (**C**). Analysis with paired two-tailed Student’s *t* test was performed in GraphPad Prism v. 9.0.

The percentage of endometrial lymphocytes with the CD3-phenotype was 26.2 ± 3.4% (mean ± SEM) in estrus and 32.6 ± 4.3% in diestrus. The mean proportion of CD3-NKp46+ cells in the endometrium across the cycle was not significantly different, being 6 ± 1% in mares during estrus and 6.5 ± 1.4% in diestrus (Figure 2A). The percentage of CD3-NKp46^+^ cells in the endometrium varied greatly between mares and ranged from 2.1% to 12.7% of the total number of lymphocytes across the cycle. There was no correlation between the percentage of CD3-NKp46^+^ cells and either mare age or mare parity.

### 3.2. Tregs

The percentage of CD4^+^ lymphocytes did not differ significantly between estrus and diestrus (9.1 ± 3.7% in estrus to 6.7 ± 3.6% in diestrus). The mean proportion of lymphocytes with CD4^+^FOXP3^+^ phenotype was 14.5 ± 5.9% in estrus and 13.7 ± 6.2% in diestrus (Figure 2B). The mean percentage of Tregs with CD4^+^FOXP3^+^CD25^+^ phenotype was 3.6 ± 2.1% in estrus and 2 ± 2% in diestrus (*p* = 0.0947, Figure 2C). There was no correlation between the percentage of CD4^+^FOXP3^+^ or CD4^+^FOXP3^+^CD25^+^ cells and either mare age or mare parity.

**Figure 3:**
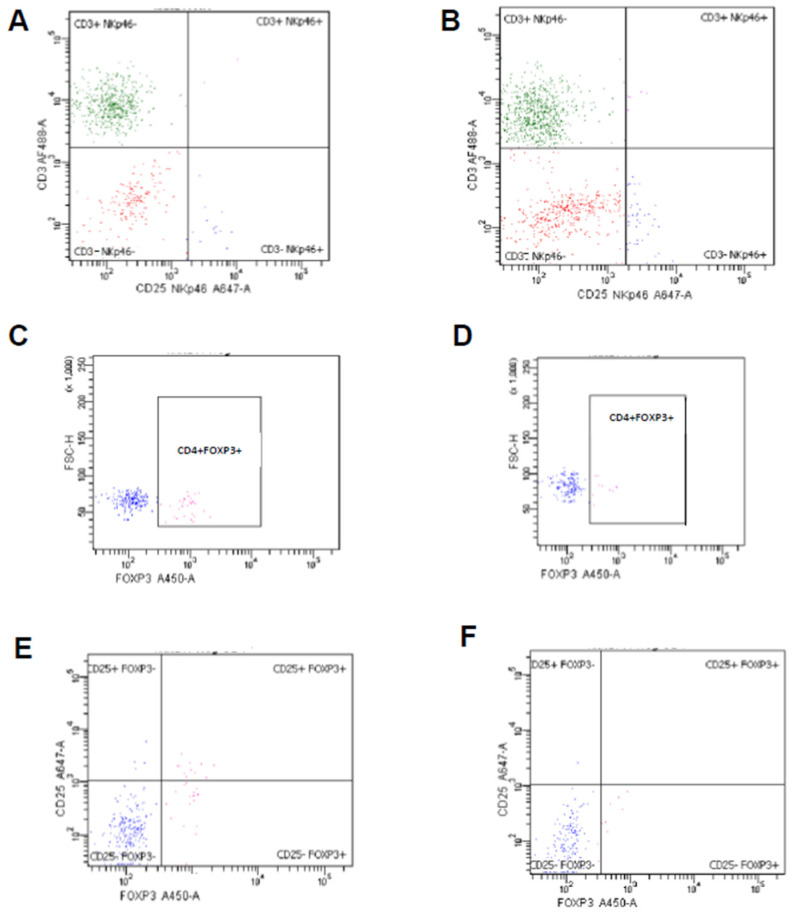
Representative images of endometrial lymphocytes expressing markers specific for NK cells and regulatory T cells (Tregs). Phenotype of endometrial lymphocytes isolated from mares in estrus (**A**,**C**,**E**) and diestrus (**B**,**D**,**F**). Lymphocytes were labeled with anti-equine CD3 and NKp46 monoclonal antibodies (**A**,**B**). For Tregs, cells were labeled with anti-equine CD4, anti-mouse FOXP3 monoclonal antibody (**C**,**D**) and additionally CD25 monoclonal antibody (**E**,**F**). Plots show cells gated on lymphocytes (**A**,**B**) and lymphocytes and CD4+ cells (**C**–**F**).

Representative images of endometrial lymphocytes expressing markers specific for NK cells and Tregs in estrus (A, C, E) and diestrus (B, D, F) are shown in Figure 3.

### 3.3. P4 Concentration

The mean P4 serum concentration measured during diestrus sample collection was 9.3 ± 2.5 ng/mL, and that measured during estrus sample collection was 0.3 ± 0.2 ng/mL. P4 concentrations of every mare during estrus and diestrus sample collection are available in Supplementary Figure S1. There was no correlation between P4 serum concentration and the percentage of Tregs or NK cells using any of the marker combinations.

## 4. Discussion

This is the first study where we showed, using specific antibodies, that CD3^−^NKp46^+^ lymphocytes and CD4^+^FOXP3^+^CD25^+^ T cells are present during estrus and diestrus in the mares’ endometrium. These lymphocytes’ phenotypes are characteristic for NK cells and Tregs across species [8,10]. Moreover, the percentages of these cells’ populations were compared across the estrous cycle, with CD4^+^CD25^+^FOXP3^+^ Tregs showing a trend of being increased in estrus compared with diestrus. NK cell numbers were similar throughout these two phases of the estrous cycle.

Expression of genetic markers of NK cells, NKp46, CD16, CD94 and CD56, were shown in a chorionic girdle and in cells surrounding the endometrial cups in mares, on days 35–46 of pregnancy [8]. Here, we showed that lymphocytes expressing NKp46 molecules are present in the equine endometrium. There was no significant difference between the percentage of NK cells with regard to the phase of the estrous cycle. Data obtained from women suggest that endometrial NK (eNK) cells are present in the endometrium of non-pregnant women, and their number but not percentage increases from the proliferative (follicular) phase to the secretory (luteal) phase of the cycle [4]. Similarly, we found no change in the percentage of NK cells across the estrous cycle in mares. These cells are thought to participate in the rebuilding of the endometrium during and after menstruation, and their phenotype is different from peripheral or decidual NK cells [4]. Cyclic changes in the mare’s endometrium differ greatly from women. There is no menstruation, no tissue fragmentation or shedding and no bleeding [19]. Thus, it is feasible to speculate that the numbers of eNK cells in mares may not change during the estrous cycle due to the absence of a biological requirement.

eNK cells are considered to have important immunoregulatory functions in the endometrium [4]. In women, eNK cells expressing NKp46 and an activating receptor NKG2D fail to produce cytokines such as IFNγ, and their cytotoxicity is presumably lower than peripheral NK cells [20,21,22]. However, after stimulation with IL-15, which supports NK cell differentiation, eNK cells start to secrete IFNγ, similar to decidual NK cells (dNK) [20,21,22]. Considering the above, it is speculated that eNK cells wait in the endometrium “just in case” of pregnancy and may be the immature form of dNK cells [4,20,21,22]. In response to pregnancy-related cytokines, they transform into dNK cells, facilitating maternal–fetal tolerance and placentation [23]. Our results showed that equine eNK cells also expressed the marker NKp46. It is feasible to speculate that in mares, similar to women, eNK cells are required for the effective preparation of the endometrium for the entering embryo and later implantation. Nevertheless, this hypothesis requires further investigation. Another explanation for the presence of eNK cells in mares’ endometrium may be their dual capability of facilitating both protection against pathogens and immunotolerance [22]. In women, in the case of viral infection and further cytokine stimulation (e.g., IL-2), the cytotoxicity of dNK cells may be restored [22,23], and it is suggested that these lymphocytes might be important for fighting virus-infected cells. Whether eNK cells may have a similar function during the estrous cycle in mares remains unknown. It is worth noting that over 80% of horses, including mares, are life-long latently infected with equine herpes virus type 1 (EHV-1) [24]; hence, the involvement of eNK cells in viral protection during pregnancy is worth further consideration.

Regulatory T cells are present in the endometrium of females of many species both during the estrous cycle and pregnancy [5,23,25]. There is still little known about their abundance and function in the mare’s reproductive tract. Existing data on Treg cells come in large part from studies on humans and mice and are focused primarily on the CD4^+^FOXP3^+^CD25^+^ cells. Due to a previous report on equine peripheral Tregs, we decided to assess two populations, CD4^+^FOXP3^+^ and CD4^+^FOXP3^+^CD25^+^ cells [9,12]. CD4^+^FOXP3^+^ cells have been identified in the endometrium of pregnant [9] and non-pregnant mares [26].

Results from other species report small numbers of Tregs in the endometrium throughout the estrous cycle [5,23,26]. Our data showed that the average percentage of CD4 T cells expressing FOXP3 in equine endometrium was higher than peripheral CD4 T cells, 14.1% (versus 2.2% in periphery [12]), and similar when compared to CD4 cells around the endometrial cups [9]. It is speculated that the proliferation of Tregs present during the follicular phase of the cycle occurs in response to a rising concentration of estradiol (E2) and an increase in chemokines which recruit Tregs into the uterus, reaching a maximum number just before ovulation [13]. This periodic increase in the percentage of endometrial Tregs may influence successful conception [5,23,26]. The proliferative effect of E2 on the peripheral population of human CD4^+^CD25^+^FOXP3^+^ cells was shown in vitro and in vivo [27,28]. Moreover, E2 increases expression of both FOXP3 and CD25 in CD4^+^ T lymphocytes [27]. Indeed, we observed a trend towards a higher percentage of CD4^+^CD25^+^FOXP3^+^ cells in the endometrial samples collected during estrus than diestrus. It might be proposed that in mares, as it is suggested in other species, E2 promotes the proliferation of CD4^+^CD25^+^FOXP3^+^ Tregs in the endometrium.

Surprisingly, no significant differences were noted for CD4^+^FOXP3^+^ Tregs. Populations of regulatory T cells are characterized based on the expression of various receptors/factors which usually determine their functions [29]. Moreover, the expression of some receptors may differ between species, with CD127, for example, being specific to humans [29]. Expression of CD25 was detected both on unstimulated [30] and stimulated equine peripheral Tregs [11]. Still, both CD4+FOXP3+ and CD4+CD25+FOXP3+ phenotypes, including those present in the uterus, characterize immunoregulatory properties [29]. In mares, the embryo descends around day 6 to 7 of pregnancy, and implantation starts 10 days later. However, a lower level of peripheral CD4^+^FOXP3^+^ Tregs was noted in mares that experience early pregnancy loss [31]. A similar phenomenon was observed in women enduring recurrent spontaneous abortion [28].

On the other hand, no changes in the amount of FOXP3^+^ cells in histological sections of the endometrium were found after intrauterine treatment with seminal plasma [30]. The authors suggest two possible hypotheses. First, during the short post-insemination period (24 h), the expansion of uterine Tregs might be limited only to local lymph nodes, or Tregs percentage increases significantly and rapidly only in response to pregnancy, presumably due to fetal antigen and hormone stimulation, as suggested by others [5,31,32]. Our results showed that the percentage of CD4^+^FOXP3^+^ lymphocytes is similar during the estrous cycle to that around the endometrial cups, that is, during pregnancy [10]. Nevertheless, the study did not determine the presence of CD4^+^CD25^+^FOXP3^+^ Tregs. Whether there are any differences in biological functions of these two Tregs’ subsets in mares’ reproductive tract remains to be determined.

We acknowledge the limitations of our study. There were only 11 mares involved in the study, similar to other studies where endometrial biopsies were taken [7,26]. Large fluctuations in Tregs and NK cell percentages between mares were observed and could not be explained by mare age or a history of a previous pregnancy. Future studies may require taking samples from larger numbers of mares, as a similar variation between Tregs percentage is observed in the endometrium of women [33].

Knowledge on the NK cells and Tregs in horses is still incomplete. Specific functional populations of these cells are not yet determined, in large part due to the lack of specific antibodies. We are aware that the use of anti-CD3 and anti-NKp46 does not allow for detailed characterization of phenotypes of NK cells within and specific to the endometrium. Especially, if similar to other species, equine eNK cells could be found to have different phenotypic characteristics from the peripheral NK cells [4]. Nevertheless, NKp46 is currently considered the steadiest marker of NK cells in mammals, and it is also conserved in equine peripheral NK cells [16]. Currently, there are no other specific markers or antibodies for equine eNK or “immune tolerance inducing type” NK cells. In order to maintain their immune functions and recognize foreign antigens presented by either virus/tumor-infected cells or trophoblasts, mammalian NK cells express killer cell immunoglobulin-like receptors (KIR) and/or killer cell lectin-like (KLR) receptors [34]. Both types of receptors bind to MHC I, molecules which are also expressed by equine invasive trophoblasts [35]. Recent research indicates that equine NK cells express functional Ly49 receptor, a member of the KLR family [36]. Moreover, Ly49 was found to be expressed in the maternal–fetal interface in mares [37]. In humans, interaction between KIR and trophoblast HLA-C promotes angiogenesis of the developing placenta. However, some variants of maternal KIRs and fetal HLA-C may lead to preeclampsia or miscarriage [38]. A variant of the Ly49B gene, which belongs to the Ly49 gene family, has been found to be absent in adults and is thus suggested to be associated with a lethal phenotype of the equine embryo, although the developmental stage of the lethality is unknown [37]. Hence, it might be hypothesized that Ly49 receptor may be one of the candidates for the “immune tolerance inducing type” NK cell markers in horses.

Similar to NK cells, Tregs can be differentiated based on the expression of specific receptors [10,11]. FOXP3 is believed to be a transcription factor determining the fate of T cells, and its expression is related to their immunosuppressive properties [10,11]. In our studies, we focused on CD4^+^ T cells, which are the major pool for the endometrial Tregs in females of other species [10,11]. Based on previous reports and due to specificity and availability, the antibodies anti-CD25 and anti-FOXP3 were used [12]. Both markers are expressed by peripheral and uterine Tregs [12,26]. More recent research speculates that endometrial Tregs, similar to eNK cells, may carry a unique phenotype which distinguishes them from peripheral populations of these cells, especially in terms of immune checkpoint genes such as programmed cell death protein 1 (PD-1) or cytotoxic T-lymphocyte-associated protein 4 (CTLA-4) [29]. Furthermore, the transcriptome of endometrial Tregs might be influenced not only by hormones such as E2, but also by subsequent pregnancies [29]. Expression of the above receptors is important for facilitating the immunosuppressive functions of Tregs in the fetal–maternal interface [23]. Hence, it would be interesting to determine whether endometrial Tregs have unique markers in comparison to peripheral ones in mares.

## 5. Conclusions

We showed that NK cells and Tregs are present in the endometrium of mares at two distinct phases of the estrus cycle. Endometrial NK cells express NKp46 receptor, a marker highly conserved among studied mammals. Within endometrial Tregs, two phenotypes have been identified, CD4^+^CD25^+^FOXP3^+^ and CD4^+^FOXP3^+^ cells. A trend towards an elevation of CD4^+^CD25^+^FOXP3^+^ Tregs during estrus was observed. Due to limited data on endometrial lymphocytes in mares, these results provide a valuable benchmark for further research that assesses their role in pregnancy and disease processes.

## Figures and Tables

**Table 1:** Antibodies used in flow cytometry analysis to determine the presence of NK cells and regulatory T cells (Tregs) in mares’ endometrium.

Antibody	Clone	Isotype	Fluorochrome	Concentration	Catalogue Number/Supplier	Reference
CD3	UC-F6G	IgG1	A647	1:100	UC Davis, Davis, CA, USA	[18]
NKp46	4F2	IgG1	A488	1:50	Wagner Laboratory, Detroit, MI, USA	[16]
CD4	CVS4	IgG1	PE	1:10	MA5-28356; Invitrogen, Waltham, MA, USA	Not applicable
CD25	15-1	IgG1	A488	1:20	Wagner Laboratory, Detroit, MI, USA	[12]
FOXP3	FJK-16	IgG2a, kappa	eFluor450	1:50	12-5773-82; eBioscience, San Diego, CA, USA	[9]

## Data Availability

The data presented in this study are available on request from the corresponding author and in the Supplementary Figure S1.

## Supplementary Materials

The following supporting information can be downloaded at: https://www.mdpi.com/article/10.3390/ani12233373/s1, Supplementary Figure S1: Serum progesterone (P4) concentrations measured in blood samples taken on the day of biopsy collection.
