# Supplementary material for: Populations of NK Cells and Regulatory T Cells in the Endometrium of Cycling Mares—A Preliminary Study
Source: Animals (Basel). 2022 Nov 30;12(23):3373. doi: 10.3390/ani12233373 (PMC9740928; doi:10.3390/ani12233373)
Supplement: Supplementary file 1 [file animals-12-03373-s001.zip › animals-2018652-supplementary.pdf]

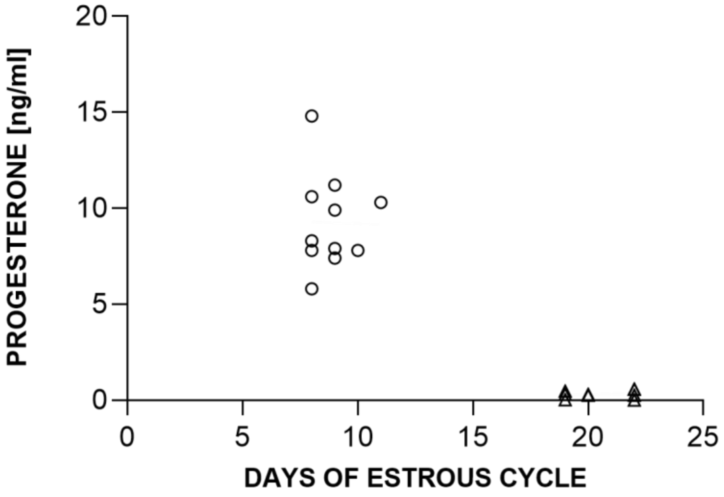

Figure S1. Serum progesterone (P4) concentrations measured in blood samples taken on the day of biopsy collection.
